# COVID-19 ARDS is characterized by higher extravascular lung water than non-COVID-19 ARDS: the PiCCOVID study

**DOI:** 10.1186/s13054-021-03594-6

**Published:** 2021-06-01

**Authors:** Rui Shi, Christopher Lai, Jean-Louis Teboul, Martin Dres, Francesca Moretto, Nello De Vita, Tài Pham, Vincent Bonny, Julien Mayaux, Rosanna Vaschetto, Alexandra Beurton, Xavier Monnet

**Affiliations:** 1 AP-HP, Service de médecine intensive-réanimation, Hôpital de Bicêtre, DMU CORREVE, Inserm UMR S_999, FHU SEPSIS, Groupe de recherche clinique CARMAS, Université Paris-Saclay, 78 rue du Général Leclerc, 94270 Le Kremlin-Bicêtre, France; 2 UVSQ, Univ. Paris-Sud, Inserm, Equipe d’Epidémiologie respiratoire intégrative, CESP, Université Paris-Saclay, 94807 Villejuif, France; 3 AP-HP, Groupe Hospitalier Universitaire APHP-Sorbonne Université, site Pitié-Salpêtrière, Service de Pneumologie, Médecine intensive Réanimation (Département R3S), Paris, France; 4 INSERM, UMRS_1158 Neurophysiologie respiratoire expérimentale et clinique, Sorbonne Université, Paris, France; 5 Università del Piemonte Orientale, Anestesia e Terapia Intensiva, Azienda Ospedaliero Universitaria ’Maggiore Della Carità”, Novara, Italy

**Keywords:** Transpulmonary thermodilution, Hemodynamic monitoring, Mechanical ventilation, SARS-CoV-2

## Abstract

**Background:**

In acute respiratory distress syndrome (ARDS), extravascular lung water index (EVLWi) and pulmonary vascular permeability index (PVPI) measured by transpulmonary thermodilution reflect the degree of lung injury. Whether EVLWi and PVPI are different between non-COVID-19 ARDS and the ARDS due to COVID-19 has never been reported. We aimed at comparing EVLWi, PVPI, respiratory mechanics and hemodynamics in patients with COVID-19 ARDS *vs.* ARDS of other origin.

**Methods:**

Between March and October 2020, in an observational study conducted in intensive care units from three university hospitals, 60 patients with COVID-19-related ARDS monitored by transpulmonary thermodilution were compared to the 60 consecutive non-COVID-19 ARDS admitted immediately before the COVID-19 outbreak between December 2018 and February 2020.

**Results:**

Driving pressure was similar between patients with COVID-19 and non-COVID-19 ARDS, at baseline as well as during the study period. Compared to patients without COVID-19, those with COVID-19 exhibited higher EVLWi, both at the baseline (17 (14–21) *vs.* 15 (11–19) mL/kg, respectively, *p* = 0.03) and at the time of its maximal value (24 (18–27) *vs.* 21 (15–24) mL/kg, respectively, *p* = 0.01). Similar results were observed for PVPI. In COVID-19 patients, the worst ratio between arterial oxygen partial pressure over oxygen inspired fraction was lower (81 (70–109) *vs.* 100 (80–124) mmHg, respectively, *p* = 0.02) and prone positioning and extracorporeal membrane oxygenation (ECMO) were more frequently used than in patients without COVID-19. COVID-19 patients had lower maximal lactate level and maximal norepinephrine dose than patients without COVID-19. Day-60 mortality was similar between groups (57% *vs.* 65%, respectively, *p* = 0.45). The maximal value of EVLWi and PVPI remained independently associated with outcome in the whole cohort.

**Conclusion:**

Compared to ARDS patients without COVID-19, patients with COVID-19 had similar lung mechanics, but higher EVLWi and PVPI values from the beginning of the disease. This was associated with worse oxygenation and with more requirement of prone positioning and ECMO. This is compatible with the specific lung inflammation and severe diffuse alveolar damage related to COVID-19. By contrast, patients with COVID-19 had fewer hemodynamic derangement. Eventually, mortality was similar between groups.

**Trial registration number and date of registration:**

ClinicalTrials.gov (NCT04337983). Registered 30 March 2020—Retrospectively registered, https://clinicaltrials.gov/ct2/show/NCT04337983.

**Supplementary Information:**

The online version contains supplementary material available at 10.1186/s13054-021-03594-6.

## Introduction

Five to 20 percent of the patients hospitalized for coronavirus disease 2019 (COVID-19) develop acute respiratory distress syndrome (ARDS) [1–4]. Numerous studies have described respiratory mechanics in COVID-19 ARDS, reporting different phenotypes [5–8] and have compared them to respiratory mechanics of non-COVID-19 ARDS patients [9–14].

The characteristics of COVID-19 in terms of extravascular lung water indexed for body weight (EVLWi) have not been described yet. EVLWi, which can be measured at the bedside through transpulmonary thermodilution [15], quantifies the thermal capacity of the lung. In non-COVID-19 ARDS, it reflects the volume of fluid contained in the interstitium and the alveoli, but also the volume of inflammatory tissue accumulated because of lung injury from various intra- and extra-pulmonary etiologies [16]. As such, it quantifies the degree of alveolar damage [17]. Calculated as the ratio of EVLWi and cardiac preload, the pulmonary vascular permeability index (PVPI) reflects the pulmonary leak [18] and indicates the degree of vascular injury during non-COVID-19 ARDS [19]. Our group has already shown that the maximal value of EVLWi and of PVPI reached during a non-COVID-19 ARDS episode are independent factors associated with mortality [20].

It has been suggested that COVID-19 and ARDS are distinct entities [21, 22]. Whether EVLWi and PVPI would indicate the specificity of COVID-19 lung injury is unclear. Also, by indicating the risk of fluid overload due to fluid infusion, EVLWi and PVPI might be used for guiding fluid therapy [16]. Therefore, comparing patients with non-COVID-19 ARDS and COVID-19 ARDS may indicate whether they require a different fluid strategy.

The primary goal of this study was to compare the levels and time course of EVLWi and PVPI in ARDS patients with and without COVID-19. We made the hypothesis that the great severity of inflammation during COVID-19 would specifically result in higher values of EVLWi and PVPI than in patients without COVID-19 [23–25]. The secondary goals were to compare these two populations in terms of lung mechanics and outcome and to describe the hemodynamic profile of critically ill patients with COVID-19.

## Methods

This observational study was conducted in intensive care units (ICUs) from three different university hospitals: Bicêtre and Pitié-Salpêtrière hospitals in Paris, France, and Maggiore Della Carità hospital in Novara, Italy. It was approved by the ethics committee of the French Intensive Care Society (CESRLF 20–25) and was registered on ClinicalTrials.gov (NCT04337983).

In the COVID-19 group, patients were consecutively included, from the first day on which COVID-19 patients started to be admitted in each ICU to October 30. Inclusion criteria were: age ≥ 18 years, presence of ARDS [26], infection by severe acute respiratory syndrome coronavirus 2 (SARS-CoV-2) confirmed by reverse transcriptase–polymerase chain reaction on a nasal swab or a tracheal aspiration, invasive mechanical ventilation, monitoring in place with transpulmonary thermodilution (PiCCO2, Pulsion Medical Systems, Feldkirchen, Germany) [27]. The exclusion criterion was the presence of an extracorporeal membrane oxygenation (ECMO) assistance at the time of inclusion, since ECMO impairs the reliability of transpulmonary thermodilution [15]. Patients were all hospitalized in standard ICUs, with trained teams and no shortage in medications or ventilators.

In the non-COVID-19 group, we retrospectively selected a similar number of ARDS patients as in the COVID-19 group, consecutively hospitalized immediately before the first COVID-19 patient. Except the absence of SARS-CoV-2 infection, the inclusion and exclusion criteria were the same as for COVID-19 patients.

### Transpulmonary thermodilution

All patients were equipped with a thermistor-tipped arterial catheter introduced through the femoral artery and an internal jugular vein catheter [15, 27]. The results obtained from three injections of cold saline boluses were averaged [28]. Besides cardiac output, transpulmonary thermodilution estimates the global end-diastolic volume indexed for body surface (index of cardiac preload) [29, 30], the global ejection fraction (index of cardiac contractility), EVLWi and PVPI. Measurements were taken as requested by the attending physicians, at least once a day in patients who improved and became stable.

In all patients, we daily collected the PiCCO2 variables corresponding to the maximum value of EVLWi and of PVPI measured within the day. The worst value they reached during the study period (EVLWi_max_ and PVPI_max_, respectively) was noted. A value of EVLWi < 10 mL/kg and a value of PVPI < 3 were considered as normal [16, 18].

### Other hemodynamic measurements

Heart rate, arterial blood pressure and the dose of catecholamines were continuously recorded. We selected values recorded at the time when the transpulmonary thermodilution values of interest were assessed. The cumulative fluid balance was collected for each patient and the mean daily fluid balance was calculated.

In COVID-19 patients, the level of N-terminal pro-brain natriuretic peptide (NT-proBNP) and high sensitivity cardiac troponin T (hs-cTnT) were also collected. Besides, the modifications of repolarization on daily electrocardiograms, new onset cardiac arrhythmias or conduction blocks were assessed. Echocardiography was performed as indicated by the attending physicians.

### Ventilatory settings and respiratory measurements

In both the COVID-19 and non-COVID-19 groups, patients received protective ventilation in a volume assist-controlled mode [31]. Tidal volume was set at 6 mL/kg of predicted body weight. Respiratory rate was adjusted to prevent hypercapnia and to avoid dynamic intrinsic positive end-expiratory pressure (PEEP). The fraction of inspired oxygen (FiO_2_) was adjusted to obtain an oxygen saturation ≥ 90%. Neuromuscular blocking agents [32], prone positioning [33], inhaled nitric oxide and ECMO [34] were used as consensually suggested [26]. If ECMO was set up after inclusion, the follow-up of the patient was stopped, as it impairs the reliability of transpulmonary thermodilution [16, 18, 20]. The most recent lung CT-scan was reviewed for reporting fibrotic lesions.

Ventilator settings, respiratory mechanics and blood gas analysis results, including the ratio of arterial partial pressure of oxygen (PaO_2_) over FiO_2_, were collected daily at the same time as transpulmonary thermodilution and other hemodynamic measurements. The compliance of the respiratory system was calculated as tidal volume/(plateau pressure—total PEEP). Ventilation-free days were calculated at 28 days. Patients who received ventilation for less than one day were considered as being ventilated 0.5 day. Deceased patients were considered as having zero ventilator-free days.

### Statistical analysis

Variables were expressed as number and proportion, mean ± standard deviation or median (interquartile range). Statistical analysis was performed using parametric (Fischer’s exact test and paired Student’s *t* test) or nonparametric tests (Mann–Whitney and Wilcoxon tests). Proportions were compared with the Pearson’s Chi-squared test or Fischer’s exact test. Univariate regression analysis was used to identify risk factors for ICU death. For comparing patients with higher and lower EVLWi and PVPI, these continuous variables were transformed in binary variables (higher or lower) according to the value defined by the receiver operating characteristic curve (ROC) as the one predicting mortality with the best Youden index.

Variables found to be significantly associated with mortality with a *p* value < 0.20 at univariate analysis were introduced into a logistic regression model. All significant variables with collinearity were excluded from the regression model. When deciding which covariates to retain as candidate predictors for the multivariable model, we considered their clinical relevance. The adjusted odds ratio (OR) of dying and the 95% confidence interval (95% CI) were calculated for all independent factors associated with mortality. A first multivariate analysis was performed by entering EVLWi_max_ as an independent factor, and a second one was performed by entering PVPI_max_ as an independent factor, as both values are correlated. A *p* value < 0.05 was considered significant. The statistical analysis was made using MedCalc 19.2.1 software (MedCalc Software Ltd, Ostend, Belgium).

## Results

### General characteristics of COVID-19 patients

Sixty patients were included in the COVID-19 group, 42 during the first hit of the outbreak in Europe (from March 1 to May 29) and 18 during the second one (from July 12 to October 30). The first symptoms of COVID-19 appeared 8 (5–12) days before ICU admission. Day-60 mortality in these COVID-19 patients was 65% (Table 1, see Additional file 1: Table S1). Lung CT-scan was available for 53 patients (88%) patients. In 25 patients, the most recent one was performed before investigation (4 (2–5) days before inclusion). In the 28 remaining patients, it was performed during investigation (8 (5–11) days after inclusion). Fibrotic lesions were reported in none of them.

**Table 1:** Comparison of demographic characteristics of COVID-19 and non-COVID-19 acute respiratory distress syndrome

Variables	COVID-19 (*N* = 60)	Non-COVID-19 (*N* = 60)	*p* value
Age (years)	64 (54–72)	62 (55–73)	0.967
Male (*n*)	46 (77)	34 (57)	**0.033**
BMI (kg/m^2^)	29.2 (26.3–33.2)	26.0 (21.8–32.0)	**0.010**
SAPS II score	40 (32–49)	55 (46–66)	** < 0.001**
SOFA total	6 (3–9)	9 (7–11)	**0.001**
SOFA respiration	2 (2–3)	3 (3–4)	**0.015**
SOFA hepatic	0 (0–0)	0 (0–1)	**0.030**
SOFA cardiovascular	2 (0–4)	4 (0–4)	**0.036**
SOFA coagulation	0 (0–1)	0 (0–1)	0.673
SOFA central nervous system	0 (0–0)	0 (0–1)	**0.002**
SOFA renal	0 (0–2)	1 (0–2)	0.094
*Medical history*			
Hypertension (*n*)	29 (48)	24 (40)	0.462
Diabetes mellitus (*n*)	24 (40)	16 (27)	0.175
COPD/asthma (*n*)	8 (13)	6 (10)	0.776
Chronic kidney disease (*n*)	10 (17)	7 (12)	0.601
Immunodepression (*n*)	13 (22)	31 (52)	**0.001**
Smoking (*n*)	8 (13)	25 (42)	**0.001**
Alcohol abuse (*n*)	6 (10)	19 (32)	**0.007**
*Adjunctive therapies*			
Prone position (*n*)	50 (83)	39 (65)	**0.037**
Sessions (*n*)	4 (1–7)	1 (0–2)	** < 0.001**
NMBA (*n*)	45 (75)	43 (72)	0.837
Corticosteroids for septic shock* (*n*)	11 (18)	47 (78)	** < 0.001**
Corticosteroids for COVID-19**	18 (30)	0 (0)	** < 0.001**
Time between corticoids start and first thermodilution	2 (1–4)***	1 (1–3.5)****	**0.628**
Inhaled nitric oxide (*n*)	6 (10)	4 (7)	0.741
ECMO (*n*)	17 (28)	7 (12)	**0.040**
Renal replacement therapy (*n*)	13 (22)	26 (43)	**0.019**
HFNC (*n*)	31 (52)	14 (23)	**0.003**
Duration of HFNC before MV (days)	3 (2–4)	1.5 (1–2)	**0.032**
NIV (*n*)	3 (5)	7 (12)	**0.322**
Duration of NIV before MV (days)	1(-)	1 (0.6–2.5)	0.555
Time from MV to TPTD (days)	1 (0–1)	0 (0–1)	**0.008**
ICU length of stay (days)	15 (8–24)	17 (9–26)	0.440
Duration of MV (days)	13 (6–22)	15 (6–23)	0.532
MV free days (days)	0 (0–3)	0 (0–8)	0.386

Bold font indicates statistical significance

Values are expressed as median (interquartile range) or *n* (%)

*ARDS* acute respiratory distress syndrome, *BMI* body mass index, *COPD* chronic obstructive pulmonary disease, *ECMO* extracorporeal membrane oxygenation, *HFNC* high-flow nasal canula, *ICU* intensive care unit, *MV* mechanical ventilation, *NIV* noninvasive ventilation, *NMBA* neuromuscular blocking agent, *SAPS* simplified acute physiologic score, *SOFA* Sequential Organ Failure Assessment, *TPTD* transpulmonary thermodilution

^*^ Hydrocortisone 200 mg/d; ** dexamethasone 6 mg/d, equivalent to hydrocortisone 160 mg/d; *** reported for the 18 patients who received corticosteroids before the set-up of the thermodilution device. **** reported for the 4 patients who received corticosteroids before the set-up of the thermodilution device

### General characteristics of non-COVID-19 patients

Non-COVID-19 ARDS patients were included between December 2018 and February 2020. In these patients, ARDS was attributed to community-acquired pneumonia in 38 (63%) patients, aspiration pneumonia in 8 (13%), pancreatitis in 8 (13%), exacerbation of chronic interstitial pneumonitis in 4 (7%) and ventilator-associated pneumonia in two patients. The delay between admission and intubation was 1 (0–2) day in COVID-19 patients and 1 (0–2) days in non-COVID-19 patients (*p* = 0.39). The delay between the first symptom and ICU admission was 1 (0–3) day in non-COVID-19 patients (*p* < 0.001 *vs.* COVID-19 patients). The delay between the first symptom and intubation was 9 (6–14) days in COVID-19 patients and 1 (0–3) day in non-COVID-19 patients (*p* < 0.001). Day-60 mortality in non-COVID-19 was 57% (*p* = 0.45 *vs.* COVID-19 patients). In patients with non-COVID-19 ARDS, the proportion of males was lower and the proportion of smokers, immunocompromised patients and alcohol abusers was higher than in patients with COVID-19 (Table 1). On admission, the SAPS II and the SOFA scores for the respiratory, cardiovascular and central nervous systems were higher in non-COVID-19 patients compared to the COVID-19 ones. COVID-19 and non-COVID-19 patients were monitored by transpulmonary thermodilution during 5 (3–10) and 9 (4–13) days, respectively, *p* = 0.008.

### Respiratory characteristics

No patient with and without COVID-19 received continuous positive airway pressure. The modalities and duration of ventilatory support are reported in Table 1. There was no significant difference between patients with and without COVID-19 in terms of baseline PaO_2_/FiO_2_ (Table 2). However, the worst PaO_2_/FiO_2_ reached during the study was significantly lower in COVID-19 than in non-COVID-19 patients (Table 2). There was no difference regarding respiratory driving pressure and compliance of the respiratory system, both at the baseline and at their nadir during the study period. However, patients with COVID-19 underwent more (83% *vs.* 65%, *p* = 0.04) and more frequent (4 *vs.* 1, *p* < 0.0001) sessions of prone positioning than patients without COVID-19. They more often received ECMO assistance (Table 1).

**Table 2:** Comparison of respiratory and hemodynamic variables of COVID-19 and non-COVID-19 acute respiratory distress syndrome

Variables	COVID-19 (*N* = 60)	Non-COVID-19 (*N* = 60)	*p* value
*Respiratory characteristics at baseline*			
PaO_2_/FiO_2_ (mmHg)	129 (97–175)	138 (96–172)	0.578
TV (mL/kg PBW)	6.0 (5.7–6.1)	6.0 (5.7–6.3)	0.341
PEEP (cmH_2_O)	13 (10–15)	12 (10–15)	0.243
Driving pressure (cmH_2_O)	13 (11–15)	13 (9–17)	0.373
Crs (mL/cmH_2_O)	31 (25–38)	31 (24–44)	0.469
*Maximal/minimal values of respiratory characteristics during TPTD monitoring*			
PaO_2_/FiO_2min_ (mmHg)	81 (70–109)	100 (80–124)	**0.024**
PEEP_max_ (cmH_2_O)	15 (12–16)	14 (12–16)	0.207
DP_max_ (cmH_2_O)	16 (15–18)	16 (12–21)	0.497
Crs_min_ (mL/cmH_2_O)	25 (21–28)	23 (16–36)	0.955
*Hemodynamic variables and CRP at baseline*			
CI (L/min/m^2^)	2.8 (2.1–3.5)	2.8 (2.5–4.2)	0.115
GEDVi (mL/m^2^)	728 (588–824)	696 (585–824)	0.559
EVLWi (mL/kg)	17 (14–21)	15 (11–19)	**0.029**
PVPI	3.5 (2.9–4.5)	2.8 (2.2–3.7)	**0.005**
GEF (%)	20 (15–24)	18 (15–23)	0.097
NE (µg/kg/min)	0.32 (0.13–0.63)	0.74 (0.49–1.45)	** < 0.001**
Lactate (mmol/L)	1.6 (1.3–2.1)	2.2 (1.2–3.5)	**0.028**
CRP (mg/L)	218 (146–295)	248 (125–321)	0.458
*Maximal/minimal values of hemodynamic variables and CRP during TPTD monitoring*			
CI_min_ (L/min/m^2^)	2.4 (2.0–2.8)	2.5 (2.0–3.0)	0.524
GEDVi_min_ (mL/m^2^)	634 (548–752)	600 (538–712)	0.278
EVLWi_max_ (mL/kg)	24 (18–27)	21 (15–24)	**0.005**
PVPI_max_	4.6 (3.8–5.7)	4.1 (3.3–4.8)	**0.006**
GEF_min_ (%)	16 (13–19)	15 (11–19)	0.258
NE_max_ (µg/kg/min)	0.51 (0.16–0.89)	1.01 (0.52–1.74)	**0.000**
Lactate_max_ (mmol/L)	2.0 (1.6–2.7)	2.7 (2.1–4.3)	**0.001**
Fluid balance (mL/day)	1016 (235–1844)	1033 (436–1715)	0.989

Bold font indicates statistical significance

Values are expressed as median (interquartile range) or *n* (%)

*ARDS* acute respiratory distress syndrome, *CI* cardiac index, *Crs* respiratory system compliance, *CRP* C-reactive protein, *DP* driving pressure, *EVLWi* extravascular lung water indexed for ideal body weight, *GEDVi* global end-diastolic volume indexed for body surface, *GEF* global ejection fraction, *NE* Norepinephrine, *PaO*_*2*_*/FiO*_*2*_ ratio of the arterial partial pressure of oxygen over inspired fraction in oxygen, *PBW* predicted body weight, *PEEP* positive end-expiratory pressure, *PVPI* pulmonary vascular permeability index, *TPTD* transpulmonary thermodilution, *TV* tidal volume

### EVLWi and PVPI

**Fig. 1:**
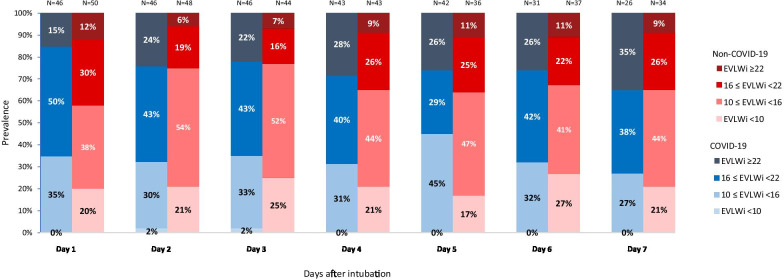
Distribution of levels of extravascular lung water index along with time in COVID-19 and non-COVID-19 acute respiratory distress syndrome

In patients with COVID-19, there was no statistical difference between survivors and non-survivors in terms of baseline cardiac index, EVLWi, PVPI and global end-diastolic volume. The evolution with time of the distribution of EVLWi values is displayed in Fig. 1 and the one of PVPI in Figure S1 (see Additional file 1). EVLWi_max_ and PVPI_max_ were reached 3 (1–4) and 3 (1–5) days after intubation, respectively. Unlike baseline values, EVLWi_max_ (24 (20–28) *vs.* 21 (16–24), *p* = 0.025) and PVPI_max_ (4.9 (4.0–6.0) *vs*. 4.2 (3.7–4.7), *p* = 0.032) were significantly higher in COVID-19 non-survivors than in survivors (see Additional file 1: Table S2).

Variables found to be significantly associated with mortality at univariate analysis (Table 3) were introduced into multivariate logistic regression analysis. Age, SAPS II were selected a priori, and we forced COVID-19 status in the model, then maximal blood lactate, the mean daily balance, the maximal driving pressure observed during transpulmonary thermodilution monitoring and EVLWi_max_ were entered in a first model. Age, the mean daily fluid balance, the maximal driving pressure and EVLWi_max_ were independently associated with Day-60 mortality (see Additional file 1: Table S3). In a second model where EVLWi_max_ was replaced by PVPI_max_, age, the mean daily fluid balance and PVPI_max_ were identified as independent predictors of mortality (see Additional file 1: Table S4).

**Table 3:** Comparison of survivors and non-survivors in the whole population of COVID-19 and non-COVID-19 acute respiratory distress syndrome

Variable	Survivors (*N* = 47)	Non-survivors (*N* = 73)	*p* value
*Demographic characteristics of ARDS with or without COVID-19*			
Age (years)	58 (44–70)	66 (59–73)	**0.028**
Male (*n*)	30 (64)	50 (68)	0.741
BMI (kg/m^2^)	27.4 (23.7–31.8)	28.8 (24.2–32.8)	0.314
SAPS II score	46 (34–58)	48 (38–66)	0.161
SOFA total	7 (4–10)	8 (4–10)	0.402
SOFA respiration	3 (2–3)	3 (2–4)	0.529
SOFA hepatic	0 (0–0)	0 (0–1)	0.091
SOFA cardiovascular	4 (0–4)	4 (0–4)	0.848
SOFA coagulation	0 (0–0)	0 (0–1)	**0.032**
SOFA central nervous system	0 (0–0)	0 (0–0)	0.102
SOFA renal	1 (0–2)	1 (0–2)	0.878
*Medical history*			
Hypertension (*n*)	18 (38)	35 (48)	0.395
Diabetes mellitus (*n*)	16 (34)	24 (33)	0.947
COPD/asthma (*n*)	5 (11)	9 (12)	0.992
Chronic kidney disease (*n*)	5 (11)	12 (16)	0.534
Immunodepression (*n*)	14 (30)	30 (41)	0.289
Smoking (*n*)	11 (23)	22 (30)	0.551
Alcohol abuse (*n*)	13 (28)	12 (16)	0.212
*Adjunctive therapies*			
Prone position (*n*)	33 (70)	56 (77)	0.562
Sessions (*n*)	1(1–4)	2 (1–5)	0.218
NMBA (*n*)	33 (70)	55 (75)	0.683
Corticosteroids (*n*)	30 (64)	46 (63)	0.918
Inhaled nitric oxide (*n*)	1 (2)	9 (12)	0.102
ECMO (*n*)	4 (9)	20 (27)	**0.022**
Renal replacement therapy (*n*)	11 (23)	28 (38)	0.132
Time from MV to TPTD (days)	1 (0–2)	0 (0–1)	**0.030**
ICU length of stay (days)	21 (15–32)	13 (6–21)	** < 0.001**
Duration of MV (days)	17 (10–28)	11 (4–19)	**0.002**
MV free days at Day 28 (days)	12 (0–18)	0 (0–0)	** < 0.001**
*Respiratory characteristics at baseline*			
PaO_2_/FiO_2_ (mmHg)	138 (109–177)	131 (93–166)	0.305
TV (mL/kg PBW)	6.0 (5.7–6.3)	5.9 (5.7–6.2)	0.135
PEEP (cmH_2_O)	12 (10–15)	12 (10–15)	0.978
DP (cmH_2_O)	12 (10–15)	13 (10–16)	0.422
Crs (mL/cmH_2_O)	33 (27–41)	30 (22–38)	0.125
*Maximal/minimal values of respiratory characteristics during TPTD monitoring*			
PaO_2_/FiO_2min_ (mmHg)	110 (85–131)	81 (70–104)	**0.000**
PEEP_max_ (cmH_2_O)	14 (12–16)	14 (12–16)	0.939
DP_max_ (cmH_2_O)	16 (12–18)	17 (14–22)	**0.015**
Crs_min_ (mL/cmH_2_O)	26 (21–32)	24 (16–27)	**0.021**
*Hemodynamic variables and CRP at baseline*			
CI (L/min/m^2^)	3.2 (2.6–4.1)	2.8 (2.1 3.7)	**0.038**
GEDVi (mL/m^2^)	696 (580–796)	739 (594–886)	0.235
EVLWi (mL/kg)	14 (10–18)	17 (13–21)	**0.024**
PVPI	3.1 (2.3–3.7)	3.3 (2.5–4.4)	0.118
GEF (%)	20 (16–25)	18 (14–23)	0.054
NE (µg/kg/min)	0.42 (0.14–0.83)	0.61 (0.29–1.29)	0.063
Lactate (mmol/L)	1.85 (1.20–2.50)	2.00 (1.40–2.80)	0.366
CRP (mg/L)	209 (133–293)	225 (123–315)	0.451
*Maximal/minimal values of TPTD variables during TPTD monitoring*			
CI_min_ (L/min/m^2^)	2.6 (2.0–3.1)	2.4 (2.0–2.7)	0.153
GEDVi_min_ (mL/m^2^)	588 (534–689)	661 (548–770)	0.042
EVLWi_max_ (mL/kg)	19 (15–24)	23 (18–26)	**0.001**
PVPI_max_	3.8 (3.3–4.7)	4.5 (3.8–5.8)	**0.003**
GEF_min_ (%)	16 (12–19)	16 (11–19)	0.359
NE_max_. (µg/kg/min)	0.40 (0.10–1.10)	0.76 (0.50–1.43)	**0.007**
Lactate_max_ (mmol/L)	2.1 (1.7–3.3)	2.5 (1.9–4.1)	0.097
Fluid balance (mL/day)	809 (35–1286)	1206 (648–2046)	**0.004**

Bold font indicates statistical significance

Values are expressed as median (interquartile range) or *n* (%)

*ARDS* acute respiratory distress syndrome, *BMI* body mass index, *CI* cardiac index, *COPD* chronic obstructive pulmonary disease, *Crs* respiratory system compliance, *CRP* C-reactive protein, *DP* driving pressure, *ECMO* extracorporeal membrane oxygenation, *EVLWi* extravascular lung water indexed for ideal body weight, *GEDVi* global end-diastolic volume indexed for body surface, *GEF* global ejection fraction, *ICU* intensive care unit, *MV* mechanical ventilation, *NE* Norepinephrine, *NMBA* neuromuscular blocking agent, PaO_2_/FiO_2_ ratio of the arterial partial pressure of oxygen over inspired fraction in oxygen, *PBW* predicted body weight, *PEEP* positive end-expiratory pressure, *PVPI* pulmonary vascular permeability index, *SAPS* simplified acute physiologic score, *SOFA* Sequential Organ Failure Assessment, *TPTD* transpulmonary thermodilution, *TV* tidal volume

Among patients with non-COVID-19 ARDS, EVLWi_max_ and PVPI_max_ were reached 3 (1–3) and 3 (1–6) days after intubation, respectively (not different from patients with COVID-19), and were higher in non-survivors compared to survivors (EVLWi_max_:22 (17–25) *vs.* 18 (14–21), respectively, *p* = 0.016; PVPI_max_:4.2 (3.4–5.0) *vs.* 3.6 (2.5–4.6), respectively, *p* = 0.042) (see Additional file 1: Table S2).

In COVID-19 patients, the delay before EVLWi_max_ was 4 (2.3–6) days in patients with high-flow nasal canula (HFNC) or noninvasive ventilation (NIV) before intubation and 4 (2–6) days in the other ones (*p* = 0.988). In non-COVID-19 patients, this delay was 2 (2–13) days in patients with HFNC/NIV before intubation and 5 (2–9) days in the other ones (*p* = 0.350).

The value of EVLWi_max_ was 24 (21–28) mL/kg in patients with HFNC/NIV before intubation and 22 (18–25) mL/kg in the other ones (*p* = 0.051). In non-COVID-19 patients, EVLWi_max_ was 21 (13–25) mL/kg in patients with HFNC/NIV before intubation and 20 (15–24) mL/kg in the other ones (*p* = 0.851). In the whole cohort of COVID-19 and non-COVID-19 patients, the delay before EVLWi_max_ was 4 (2–6) days in patients with HFNC/NIV before intubation and 5 (2–8) days in the other ones (*p* = 0.275).

In COVID-19 patients, EVLWi_max_ was not different between patients who received corticosteroids, whatever the dose (160 mg/d equivalent of hydrocortisone if indicated for COVID-19 or 200 mg/d hydrocortisone if indicated for septic shock) (24 (21–28) mL/kg vs. 22 (17–27) mL/kg, respectively, *p* = 0.482). In the whole cohort of COVID-19 and non-COVID-19 patients, the value of EVLWi_max_ was 23 (17–27) mL/kg in in patients with HFNC/NIV before intubation and 21 (15–24) mL/kg in the other ones (*p* = 0.039).

**Fig. 2:**
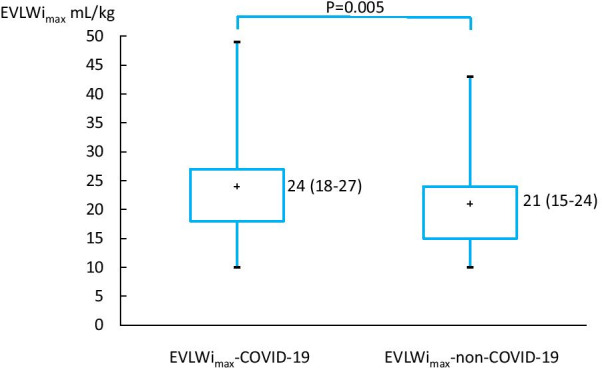
Levels of maximum value of extravascular lung water index in COVID-19 and non-COVID-19 acute respiratory distress syndrome

Compared to patients without COVID-19, patients with COVID-19 had significantly higher EVLWi (17 (14–21) *vs.* 15 (11–19), *p* = 0.029) and PVPI (3.5 (2.9–4.5) *vs.* 2.8 (2.2–3.7), *p* = 0.005) at baseline (Table 2). EVLWi_max_ (24 (18–27) *vs.* 21 (15–24), *p* = 0.005) (Table 2, Fig. 2) and PVPI_max_ (4.6 (3.8–5.7) *vs.* 4.1 (3.3–4.8), *p* = 0.006) (Table 2, see Additional file 1: Figure S2) were worse in COVID-19 than in non-COVID-19 patients. The number of days spent with EVLWi > 21 mL/kg was 1 (0–2) in COVID-19 patients and 0 (0–1) in patients without COVID-19 (*p* = 0.008). This was also the case for the number of days spent with PVPI > 4.2. The comparison between higher *vs.* lower EVLWi_max_ (EVLWi_max_ ≥ 21 and < 21 mL/kg based on etiologies and other variables are presented in Table S5 (see Additional file 1).

### Hemodynamic variables

In patients with COVID-19, echocardiography was performed within the first three days in 46 (77%) patients. Pericardial effusion occurred in 3 (5%) of these patients. The left ventricular ejection fraction was 55 (50–60) %. It was < 40% in 5 (18%) COVID-19 patients. *Acute cor pulmonale* was observed in four patients. The value of hs-cTnT was > 28 ng/L at least once during the ICU stay in 32 (53%) COVID-19 patients, among whom 22 (69%) died. The value of NT-proBNP reached a value > 5000 pg/mL at least one day during the ICU stay in 17 (28%) COVID-19 cases, among whom 12 (71%) died. Changes in the electrocardiogram repolarization, new atrial fibrillation or new conduction block appeared in 11 (18%), 8 (13%) and 6 (10%) COVID-19 patients, respectively.

Patients without COVID-19 had a higher cardiovascular SOFA score on admission (Table 1), a higher maximal dose of norepinephrine and a higher maximal blood lactate level than patients with COVID-19 (Table 2). The fluid balance was similar between groups (Table 2). Among the 32 of 60 (53%) COVID-19 patients who underwent CT pulmonary angiography, pulmonary embolism was detected in 12 (37%) of them, representing 20% of the COVID-19 population. Among the 25 (42%) patients without COVID-19 who underwent CT pulmonary angiography, pulmonary embolism was detected in 1 (4%) of them, representing 2% of the non-COVID-19 population (*p* < 0.0001). Considering both patients with and without COVID-19, EVLWi_max_ was not different between patients with and without pulmonary embolism.

## Discussion

In our study, which compared ARDS patients with and without COVID-19, we found that both the baseline and maximal levels of EVLWi and of PVPI reached during the study period were higher in patients with COVID-19. Although the baseline PaO_2_/FiO_2_ was similar between the two groups, the worst PaO_2_/FiO_2_ ratio reached during the study period was lower in patients with COVID-19, and they received prone positioning sessions and ECMO assistance more often. Despite lower severity scores and less severe hemodynamic and respiratory failures on admission in patients with COVID-19, the Day-60 mortality was similar between groups.

Our results suggest that the impairment of blood gas exchange was worse in ARDS patients with COVID-19 than in those patients with non-COVID-19 ARDS. This was indicated, in COVID-19 patients, by the lower worst PaO_2_/FiO_2_ reached during the ARDS episode, the more frequent need for prone positioning, with more sessions in prone position, and the higher number of ECMO that were set up. However, the respiratory driving pressure and compliance of the respiratory system were similar, for the baseline as for the worst reached values. This is in agreement with some reports, suggesting no specificity of that form of COVID-19 in terms of lung mechanisms [10, 11, 14]. While the differences described in the early phase of the pandemics might have been overestimated [6, 13, 35], our data confirm that at same driving pressure, oxygenation was worse in COVID-19 than in non-COVID-19 ARDS. This is in line with a recent matched study [13]. The identical level of lung compliance between COVID-19 and non-COVID-19 patients, along with the similar lung recruitability that has also been reported [11, 36], suggests that the respiratory management should not typically differ between both populations. Of note, we could not identify fibrosis on the most recent lung CT-scan performed in our patients. We cannot exclude that such a fibrosis, which might occur early in the course of the disease [37], would induce a different pattern of lung mechanics.

To our knowledge, our study is the first that reports the characteristics of COVID-19 ARDS regarding EVLWi and PVPI. In parallel with the respiratory severity but unlike respiratory mechanics, EVLWi and PVPI at the baseline and the maximal values they reached were higher in COVID-19 than in patients without COVID-19. The higher number of days spent with EVLWi > 21 mL/kg and PVPI > 4.2 also supports the higher respiratory severity of ARDS in COVID-19 as compared to those without COVID-19.

Extravascular lung water index quantifies the volume of inflammatory fluid and tissue accumulated during lung injury and is directly related to the severity of the alveolar damage [17]. Our observation that EVLWi is higher in COVID-19 than in non-COVID-19 ARDS suggests the larger extension of lung injury in the former ones. It is in accordance with the high level of lung inflammation [23–25] and the high degree of diffuse alveolar damage [38] that have been specifically observed in COVID-19 pneumonia. It may also bring arguments to those who claim that COVID-19 is a specific entity [21, 22], with different pathologies than non-COVID-19 ARDS, which is a heterogeneous syndrome [39].

The hemodynamic severity of patients with COVID-19 was less marked during the study period than in patients without COVID-19. The maximal level of lactate, the maximal dose of norepinephrine was lower. Less patients received corticosteroids for the reason of septic shock. The lower number of patients requiring renal replacement therapy even indicates the lower incidence of multi-organ dysfunction in COVID-19 than in those without COVID-19. However, the similar mortality between both groups suggests that, during COVID-19 ARDS, the respiratory severity overcomes the benefit of the less severe circulatory failure.

In line with this, although the levels of EVLWi_max_ and of PVPI_max_ predict outcome in ARDS [20, 40–43], the lower hemodynamic severity in COVID-19 patients likely compensated the fact that EVLWi_max_ and PVPI_max_ were higher in this group. It remains that, when merging both groups with and without COVID-19, EVLWi_max_ and PVPI_max_ remained independently associated with outcome at multivariate logistic regression. Of note, severity scores at admission were lower in COVID-19 patients, confirming previous observations [13, 14]. This reflects the less marked hemodynamic impairment and likely also the fact that more patients were under high-flow oxygen, with no mechanical ventilation and sedation at baseline. This suggests that, in this specific setting, severity scores on admission fail to predict mortality.

Despite hemodynamic impairment was less marked in patients with COVID-19 than those without COVID-19, the level of hs-cTnT was elevated in a large proportion of the former, as observed in a large report [44]. As previously observed also, though it was associated with mortality [44, 45], this was not associated with significant left ventricular systolic dysfunction [46–49]. Of course, this comparison is impeded by the fact that hs-cTnT levels and cardiac function were not reported in non-COVID-19 patients.

Of note, animal studies have suggested that pulmonary vascular obstruction tends to impede the detection of EVLWi by transpulmonary thermodilution because it excludes some lung regions from the diffusion of the cold bolus [50–52]. COVID-19 is characterized by a higher incidence of pulmonary embolism compared to non-COVID-19 ARDS [53–55], as we observed in our study. Microthrombosis is also frequently observed [56]. These phenomena may have led to an underestimation of EVLWi in our COVID-19 patients. Nevertheless, EVLWi and PVPI were similar among patients with and without pulmonary embolism in the whole population.

As shown in Fig. 1 and Figure S1 (see Additional file 1), whichever the day of evolution, the level of EVLWi and PVPI was heterogeneous among patients in both groups. Then, in COVID-19, the recommendation to maintain a restrictive fluid strategy [57] may not be appropriate for all patients at any time of the disease. For instance, in some patients with acute circulatory failure at some days, the relatively low level of EVLWi and PVPI indicates a low risk of fluid administration. This suggests that monitoring EVLWi and PVPI may help individualize and optimize fluid therapy according to the risk of fluid infusion and follow the evolution of this risk over time.

Some limitations of our study deserve consideration. First, due to the number of patients, no matching could be performed between patients with and without COVID-19. However, a matching on severity at baseline may have hidden the fact that the lower hemodynamic severity compensated the higher respiratory one, leading to a similar mortality rate in both groups. Second, not all our patients received CT pulmonary angiography in both groups. Nevertheless, EVLWi_max_ was not different between patients with and without pulmonary embolism both in patients with and without COVID-19. Third, due to the lack of reliability of transpulmonary thermodilution under ECMO, data were not collected during the study period when ECMO was used.

## Conclusions

Our study shows that COVID-19 ARDS had similar respiratory mechanics as non-COVID-19 ARDS. However, the baseline and worst reached levels of EVLWi and PVPI were higher in patients with COVID-19, which was in accordance with the higher severity of the disease in terms of gas exchange alteration, prone positioning and ECMO use. It likely indicated the more marked lung tissue inflammation and diffuse alveolar damage of such a lung injury. The hemodynamic impairment was less marked in COVID-19 than in non-COVID-19 patients and, eventually, day-60 mortality was similar between groups. Finally, the heterogeneity of EVLWi and PVPI values observed in patients with COVID-19 suggest that the fluid strategy should not be homogeneously restrictive but should be individualized.

## Supplementary Information

**Additional file 1**. Supplementary information on further results.

## Data Availability

The datasets used and/or analyzed during the current study are available from the corresponding author on reasonable request.

## Abbreviations

ARDS: Acute respiratory distress syndrome
COVID-19: Coronavirus disease 2019
ECMO: Extracorporeal membrane oxygenation
FiO_2_: Fraction of inspired oxygen
ICU: Intensive care unit
HFNC: High-flow nasal canula
hs-cTnT: High sensitivity cardiac troponin T
NIV: Noninvasive ventilation
NT-proBNP: N-terminal pro-brain natriuretic peptide
PaO_2_: Arterial partial pressure of oxygen
PEEP: Positive end-expiratory pressure
SAPS: Simplified acute physiologic score
SOFA: Sequential Organ Failure Assessment
