# Supplementary material for: COVID-19 ARDS is characterized by higher extravascular lung water than non-COVID-19 ARDS: the PiCCOVID study
Source: Crit Care. 2021 Jun 1;25:186. doi: 10.1186/s13054-021-03594-6 (PMC8169440; doi:10.1186/s13054-021-03594-6)
Supplement: Supplementary file 1 — Additional file 1. Supplementary information on further results. [file 13054_2021_3594_MOESM1_ESM.docx]

**COVID-19 ARDS is characterized by higher extravascular lung water than non-COVID-19 ARDS. The PiCCOVID study**

Rui SHI, MD^1^; Christopher LAI, MD^1^; Jean-Louis TEBOUL, MD, PhD^1^; Martin DRES, MD, PhD^3,4^; Francesca MORETTO, MD^1^, Nello DE VITA, MD^5^; Tài PHAM, MD, PhD^1,2^; Vincent BONNY, MD^3,4^, Julien MAYAUX, MD^3,4^; Rosanna VASCHETTO, MD, PhD^5^; Alexandra BEURTON, MD, PhD^3,4^; Xavier MONNET MD, PhD^1^.

1. Université Paris-Saclay, AP-HP, Service de médecine intensive-réanimation, Hôpital de Bicêtre, DMU CORREVE, Inserm UMR S_999, FHU SEPSIS, Groupe de recherche clinique CARMAS, Le Kremlin-Bicêtre, France

Université Paris-Saclay, UVSQ, Univ. Paris-Sud, Inserm, Equipe d’Epidémiologie respiratoire intégrative, CESP, 94807, Villejuif, France

1. AP-HP, Groupe Hospitalier Universitaire APHP-Sorbonne Université, site Pitié-Salpêtrière, Service de Pneumologie, Médecine intensive Réanimation (Département R3S), Paris, France
2. Sorbonne Université, INSERM, UMRS_1158 Neurophysiologie respiratoire expérimentale et clinique, Paris, France
3. Università del Piemonte Orientale, Anestesia e Terapia Intensiva, Azienda Ospedaliero Universitaria "Maggiore Della Carità", Novara, Italy.

**Correspondence and requests for reprints should be addressed to :** Prof. Xavier MONNET, Service de médecine intensive-réanimation, Hôpital de Bicêtre, 78 rue du Général Leclerc, Le Kremlin-Bicêtre, 94270 France. E-mail: [xavier.monnet@aphp.fr](mailto:xavier.monnet@aphp.fr)

Table of contents

[**Table S1.** Comparison of demographic characteristics of COVID-19 and non-COVID-19 acute respiratory distress syndrome 3](#_Toc70342341)

[**Table S2.** Comparison of respiratory and haemodynamic variables of COVID-19 and non-COVID-19 acute respiratory distress syndrome 4](#_Toc70342342)

[**Table S3.** Multivariate logistic regression analysis with Day-60 mortality as the dependent factor and extravascular lung water index as an independent factor. 5](#_Toc70342343)

[**Table S4.** Multivariate logistic regression analysis with Day-60 mortality as the dependent factor and pulmonary vascular permeability index as an independent factor 6](#_Toc70342344)

[**Table S5.** Comparison of demographic characteristics of COVID-19 and non-COVID-19 acute respiratory distress syndrome based on higher *vs.* lower extravascular lung water index. 7](#_Toc70342345)

[**Figure S1.** Distribution of levels of pulmonary vascular permeability index along with time in COVID-19 and non-COVID-19 acute respiratory distress syndrome. 9](#_Toc70342346)

[**Figure S2.** Levels of maximum value of pulmonary vascular permeability index in COVID-19 and non-COVID-19 acute respiratory distress syndrome. 10](#_Toc70342347)

# **Table S1.** Comparison of demographic characteristics of COVID-19 and non-COVID-19 acute respiratory distress syndrome

| Variables | COVID-19 | | | Non-COVID-19 | | |
| --- | --- | --- | --- | --- | --- | --- |
|  | Survivors  (N = 21) | Non-survivors  (N = 39) | *p*-value | Survivors  (N = 26) | Non-survivors  (N = 34) | *p*-value |
| Age (years) | 58 (42-68) | 66 (59-73) | **0.041** | 59 (45-74) | 66 (58-73) | 0.293 |
| Male (n) | 15 (71) | 31 (79) | 0.701 | 15 (58) | 19 (56) | 0.902 |
| BMI (kg/m^2^) | 29.6 (26.6-33.7) | 29.2 (25.6-32.8) | 0.664 | 24.0 (20.9-29.9) | 27.5 (22.4-32.4) | 0.136 |
| SAPS II score | 34 (31-46) | 44 (34-57) | 0.054 | 52 (44-64) | 57 (47-74) | 0.266 |
| SOFA total | 5 (3-7) | 7 (3-10) | 0.191 | 8 (7-12) | 9 (6-11) | 0.725 |
| SOFA respiration | 2 (2-3) | 3 (2-4) | 0.208 | 3 (3-4) | 3 (2-4) | 0.604 |
| SOFA hepatic | 0 (0-0) | 0 (0-0) | 0.114 | 0 (0-0) | 0 (0-1) | 0.167 |
| SOFA cardiovascular | 0 (0-4) | 2 (0-4) | 0.540 | 4 (1-4) | 4 (0-4) | 0.692 |
| SOFA coagulation | 0 (0-0) | 0 (0-1) | **0.026** | 0 (0-1) | 0 (0-1) | 0.302 |
| SOFA central nervous system | 0 (0-0) | 0 (0-0) | 0.072 | 0 (0-1) | 0 (0-0) | 0.596 |
| SOFA renal | 0 (0-1) | 0 (0-2) | 0.307 | 1 (0-3) | 1 (0-2) | 0.631 |
| **Medical history** |  |  |  |  |  |  |
| Hypertension (n) | 10 (48) | 19 (49) | 0.850 | 8 (31) | 16 (47) | 0.312 |
| Diabetes mellitus (n) | 8 (38) | 16 (41) | 0.956 | 8 (31) | 8 (24) | 0.739 |
| COPD/asthma (n) | 3 (14) | 5 (13) | 0.811 | 2 (8) | 4 (12) | 0.931 |
| Chronic kidney disease (n) | 3 (14) | 7 (18) | 1.000 | 2 (8) | 5 (15) | 0.665 |
| Immunodepression (n) | 3 (14) | 10 (26) | 0.490 | 11 (42) | 20 (59) | 0.314 |
| Smoking (n) | 1 (5) | 7 (18) | 0.301 | 10 (38) | 15 (44) | 0.862 |
| Alcohol abuse (n) | 4 (19) | 2 (5) | 0.207 | 9 (35) | 10 (29) | 0.881 |
| **Adjunctive therapies** |  |  |  |  |  |  |
| Prone position (n) | 17 (81) | 33 (85) | 1.000 | 16 () | 23 (68) | 0.827 |
| Sessions (n) | 3 (1-6) | 4 (2-7) | 0.340 | 1(0-2) | 1(0-3) | 0.765 |
| NMBA (n) | 16 (76) | 29 (74) | 0.876 | 17 (65) | 26 (76) | 0.512 |
| Corticosteroids for septic shock* (n) | 6 (29) | 5 (13) | 0.169 | 19 (73) | 28 (82) | 0.584 |
| Corticosteroids for COVID-19** | 5 (24) | 13 (33) | 0.560 | 0 (0) | 0 (0) | - |
| Inhaled nitric oxide (n) | 0 (0) | 6 (15) | 0.149 | 1 (4) | 3 (9) | 0.808 |
| ECMO (n) | 3 (14) | 14 (36) | 0.141 | 1 (4) | 6 (18) | 0.213 |
| Renal replacement therapy (n) | 2 (10) | 9 (23) | 0.345 | 9 (35) | 17 (50) | 0.353 |
| HFNC (n) | 12 (57) | 19 (49) | 0.595 | 4 (15) | 10 (29) | 0.235 |
| Duration of HFNC before MV (days) | 3 (2-5) | 3 (2-4) | 1.000 | 1 (0.8-3) | 2 (1-2) | 0.518 |
| NIV (n) | 1 (5) | 2 (5) | 1.000 | 2 (8) | 5 (15) | 0.688 |
| Duration of NIV before MV (days) | - | - | - | 0.8 (-) | 1 (0.9-3.3) | 0.329 |
| Time from MV to TPTD (days) | 1 (1-2) | 1 (0-1) | 0.120 | 0 (0-1) | 0 (0-1) | 0.071 |
| ICU length of stay (days) | 21 (13-31) | 13 (7-19) | **0.012** | 22 (17-33) | 12 (4-22) | **0.001** |
| Duration of MV (days) | 17 (10-28) | 11 (4-18) | **0.034** | 18 (10-27) | 11 (3-22) | **0.032** |
| MV free days at Day 28 (days) | 11 (0-19) | 0 (0-0) | **<0.001** | 11 (1-18) | 0 (0-0) | **<0.001** |

Values are expressed as median (interquartile range) or n (%). Bold font indicates statistical significance.

* Hydrocortisone 200mg/d; ** dexamethasone 6 mg/d, equivalent to hydrocortisone 160 mg/d; ARDS: acute respiratory distress syndrome; BMI: body mass index; COPD: chronic obstructive pulmonary disease; ECMO: extracorporeal membrane oxygenation; HFNC: high-flow nasal canula, ICU: intensive care unit; MV: mechanical ventilation; NIV: non-invasive ventilation, NMBA: neuromuscular blocking agent; SAPS: simplified acute physiologic score; SOFA: Sequential Organ Failure Assessment; TPTD: transpulmonary thermodilution.

| **Table S2.** Comparison of respiratory and hemodynamic variables of COVID-19 and non-COVID-19 acute respiratory distress syndrome | | | | | | |
| --- | --- | --- | --- | --- | --- | --- |
| Variables | COVID-19 | | | Non-COVID-19 | | |
|  | Survivors  (N = 21) | Non-survivors  (N = 39) | *p*-value | Survivors  (N = 26) | Non-survivors  (N = 34) | *p-*value |
| **Respiratory characteristics at baseline** | | | | | | |
| PaO_2_/FiO_2_ (mmHg) | 136 (111-178) | 120 (83-160) | 0.152 | 138 (96-168) | 133 (95-176) | 0.982 |
| TV (mL/kg PBW) | 5.9 (5.7-6.1) | 5.9 (5.7-6.1) | 1.000 | 6.1 (6.0-6.6) | 5.9 (5.6-6.2) | **0.016** |
| PEEP (cmH_2_O) | 12 (10-15) | 13 (10-15) | 0.925 | 12 (10-15) | 12 (10-15) | 0.963 |
| DP (cmH_2_O) | 13 (11-15) | 13 (11-16) | 0.516 | 12 (10-16) | 13 (9-17) | 0.747 |
| Crs (mL/cmH_2_O) | 33 (27-39) | 31 (23-38) | 0.310 | 33 (27-45) | 29 (21-44) | 0.334 |
| **Maximal/minimal values of respiratory characteristics during TPTD monitoring** | | | | | | |
| PaO_2_/FiO_2min_ (mmHg) | 110 (89-126) | 77 (62-93) | **0.000** | 109 (80-132) | 93 (79-112) | 0.197 |
| PEEP_max_ (cmH_2_O) | 15 (12-16) | 15 (12-16) | 0.667 | 14 (12-16) | 13 (12-16) | 0.439 |
| DP_max_ (cmH_2_O) | 16 (14-17) | 17 (16-20) | **0.012** | 15 (12-20) | 17 (13-23) | 0.148 |
| Crs_min_ (mL/cmH_2_O) | 26 (24-28) | 24 (19-27) | **0.036** | 26 (18-38) | 21 (15-30) | 0.107 |
| **Hemodynamic variables and CRP at baseline** | | | | | | |
| CI (L/min/m^2^) | 3.3 (2.4-3.6) | 2.7 (2.0-3.4) | 0.183 | 3.0 (2.7-4.8) | 2.8 (2.3-4.0) | 0.096 |
| GEDVi (mL/m^2^) | 697 (568-813) | 739 (632-889) | 0.216 | 695 (593-770) | 729 (549-886) | 0.698 |
| EVLWi (mL/kg) | 15 (13-19) | 18 (14-22) | 0.130 | 14 (10-17) | 15 (13-19) | 0.174 |
| PVPI | 3.4(2.6-3.9) | 3.6 (3.0-4.7) | 0.196 | 2.7 (2.0-3.6) | 2.9 (2.2-3.8) | 0.340 |
| GEF (%) | 23 (19-26) | 19 (15-24) | **0.046** | 19 (16-23) | 18 (14-23) | 0.333 |
| NE (µg/kg/min) | 0.17 (0.09-0.49) | 0.34 (0.16-0.70) | 0.144 | 0.55 (0.23-1.25) | 1.12 (0.61-1.69) | 0.056 |
| Lactate (mmol/L) | 1.5 (1.2-2.0) | 1.9 (1.4-2.5) | **0.037** | 2.3 (1.2-3.5) | 2.1 (1.3-3.6) | 0.866 |
| CRP (mg/L) | 218 (156-271) | 211 (117-314) | 0.580 | 193 (127-316) | 264 (124-326) | 0.545 |
| **Maximal/minimal values of haemodynamic variables and CRP during TPTD monitoring** | | | | | | |
| CI_min_ (L/min/m^2^) | 2.6 (2.0-3.1) | 2.4 (2.0-2.7) | 0.248 | 2.7 (2.1-2.9) | 2.4 (1.9-3.1) | 0.442 |
| GEDVi_min_ (mL/m^2^) | 602 (527-699) | 669 (552-780) | 0.113 | 586 (544-649) | 657 (532-750) | 0.200 |
| EVLWi_max_ (mL/kg) | 21 (16-24) | 24 (20-28) | **0.025** | 18 (14-21) | 22 (17-25) | **0.016** |
| PVPI_max_ | 4.2 (3.7-4.7) | 4.9 (4.0-6.0) | **0.032** | 3.6 (2.5-4.6) | 4.2 (3.4-5.0) | **0.042** |
| GEF_min_ (%) | 19 (14-20) | 16 (13-19) | 0.192 | 15 (12-18) | 14 (10-19) | 0.720 |
| NE_max_ (µg/kg/min) | 0.19 (0.09-0.65) | 0.59 (0.28-0.94) | **0.031** | 0.74 (0.19 -1.21) | 1.18 (0.73-2.87) | **0.021** |
| Lactate_max_ (mmol/L) | 2.0 (1.5-2.3) | 2.2 (1.7-3.2) | 0.121 | 2.4 (2.0-4.0) | 3.1 (2.2-6.1) | 0.170 |
| Fluid balance (mL/day) | 903 (19-1790) | 1016 (648-1844) | 0.278 | 809 (35-1115) | 1398 (679-2195) | **0.003** |

Values are expressed as median (interquartile range) or n (%). Bold font indicates statistical significance.

ARDS: acute respiratory distress syndrome; CI: cardiac index; Crs: respiratory system compliance; CRP: C-reactive protein; DP: driving pressure; EVLWi: extravascular lung water indexed for ideal body weight; GEDVi: global end-diastolic volume indexed for body surface; GEF: global ejection fraction; NE: Norepinephrine; PaO2/FiO2:ratio of the arterial partial pressure of oxygen over inspired fraction in oxygen; PBW: predicted body weight; PEEP: positive end-expiratory pressure; PVPI: pulmonary vascular permeability index; TPTD: transpulmonary thermodilution; TV: tidal volume.

# **Table S3.** Multivariate logistic regression analysis with Day-60 mortality as the dependent factor and extravascular lung water index as an independent factor.

| Variable | Odds ratio | 95% CI | p-value |
| --- | --- | --- | --- |
| Age (years) | 1.0390 | 1.0026-1.0768 | **0.0357** |
| COVID-19 status | 1.1987 | 0.4218-3.4072 | 0.7338 |
| SAPS II | 1.0041 | 0.9725-1.0367 | 0.8015 |
| Maximal blood lactate (mmol/L) | 1.0176 | 0.8129-1.2739 | 0.5295 |
| Daily fluid balance (mL/day) | 1.0007 | 1.0002-1.0012 | **0.0109** |
| Maximal driving pressure (cmH_2_O) | 1.1197 | 1.0116-1.2394 | **0.0291** |
| EVLWi_max_ (mL/kg) | 1.1296 | 1.0316-1.2369 | **0.0085** |

ARDS: acute respiratory distress syndrome; SAPS II: simplified acute physiology score II; EVLWi: extravascular lung water index. Bold font indicates statistical significance.

# **Table S4.** Multivariate logistic regression analysis with Day-60 mortality as the dependent factor and pulmonary vascular permeability index as an independent factor

| Variable | Odds ratio | 95% CI | p-value |
| --- | --- | --- | --- |
| Age (years) | 1.0469 | 1.0108-1.0842 | **0.0105** |
| COVID-19 status | 1.2514 | 0.4453-3.5167 | 0.6706 |
| SAPS II | 1.0040 | 0.9730-1.0361 | 0.8018 |
| Maximal blood lactate (mmol/L) | 1.0568 | 0.8464-1.3195 | 0.6256 |
| Daily fluid balance (mL/day) | 1.0007 | 1.0002-1.0012 | **0.0084** |
| Maximal driving pressure (cmH_2_O) | 1.0888 | 0.9782-1.2118 | 0.1195 |
| PVPI_max_ | 1.6757 | 1.0870-2.5833 | **0.0194** |

ARDS: acute respiratory distress syndrome; SAPS II: simplified acute physiology score II; PVPI: pulmonary vascular permeability index. Bold font indicates statistical significance.

| **Table S5.** Comparison of demographic characteristics of COVID-19 and non-COVID-19 acute respiratory distress syndrome based on higher *vs.* lower maximal value of extravascular lung water index. | | | | | | |
| --- | --- | --- | --- | --- | --- | --- |
| Variable | COVID-19 (N=60) | | | Non-COVID-19 (N=60) | | |
|  | EVLWi_max_≥ 21  (N=41) | EVLWi_max_<21 (N=19) | p-value | EVLWi_max_≥ 21  (N=30) | EVLWi_max_<21 (N=30) | p-value |
| Age (years) | 63 (56-72) | 65 (50-70) | 0.674 | 60 (51-71) | 67 (58-74) | 0.183 |
| Male (n) | 30 (73) | 16 (84) | 0.540 | 13 (43) | 21 (70) | 0.068 |
| BMI (kg/m^2^) | 29.9 (26.7-33.5) | 27.8 (23.2-31.8) | 0.127 | 25.2 (22.4-30.5) | 27.6 (22.9-32.2) | 0.609 |
| SAPS II score | 38 (30-49) | 46 (35-59) | 0.160 | 55 (42-64) | 55 (48-71) | 0.188 |
| SOFA total | 5 (3-8) | 8 (4-10) | **0.038** | 8 (4-10) | 10 (8-12) | **0.002** |
| SOFA respiration | 2 (2-3) | 3 (2-4) | 0.383 | 3 (2-4) | 3 (3-4) | 0.450 |
| SOFA hepatic | 0 (0-0) | 0 (0-0) | 0.216 | 0 (0-1) | 0 (0-0) | 0.352 |
| SOFA cardiovascular | 0 (0-4) | 4 (0-4) | 0.056 | 3 (0-4) | 4 (3-4) | **0.021** |
| SOFA coagulation | 0 (0-1) | 0 (0-1) | 0.784 | 0 (0-1) | 0 (0-2) | 0.460 |
| SOFA central nervous system | 0 (0-0) | 0 (0-0) | 0.564 | 0 (0-0) | 0 (0-1) | 0.212 |
| SOFA renal | 0 (0-2) | 1 (0-1) | 0.770 | 0 (0-1) | 2 (1-3) | **0.001** |
| Hypertension (n) | 20 (49) | 9 (47) | 0.860 | 12 (40) | 12 (40) | 0.792 |
| Diabetes mellitus (n) | 19 (46) | 5 (26) | 0.234 | 8 (27) | 8 (27) | 0.770 |
| COPD/asthma (n) | 5 (12) | 3 (16) | 0.978 | 2 (7) | 4 (13) | 0.667 |
| Chronic kidney disease (n) | 9 (22) | 1 (5) | 0.215 | 4 (13) | 3 (10) | 1.000 |
| Immunosuppression (n) | 10 (24) | 3 (16) | 0.678 | 15 (50) | 16 (53) | 1.000 |
| Smoking (n) | 4 (10) | 4 (21) | 0.430 | 13 (43) | 12 (40) | 1.000 |
| Alcohol abuse (n) | 4 (10) | 2 (11) | 0.711 | 8 (27) | 11 (37) | 0.579 |
| Prone position (n) | 38 (93) | 12 (63) | **0.013** | 21 (70) | 18 (60) | 0.588 |
| Sessions (n) | 5 (2-7) | 2 (1-5) | **0.026** | 1 (0-4) | 1 (0-2) | 0.213 |
| NMBA (n) | 34 (83) | 10 (53) | **0.031** | 24 (80) | 19 (63) | 0.252 |
| Corticosteroids (n) | 23 (56) | 6 (32) | 0.136 | 24 (80) | 22 (73) | 0.760 |
| Inhaled nitric oxide (n) | 3 (7) | 3 (16) | 0.579 | 4 (13) | 0 (0) | 0.121 |
| ECMO (n) | 12 (29) | 5 (26) | 0.943 | 6 (20) | 1 (3) | 0.108 |
| Renal replacement therapy (n) | 6 (15) | 5 (26) | 0.466 | 12 (40) | 14 (47) | 0.795 |
| Time from MV to TPTD (days) | 1 (0-1) | 1 (1-3) | **0.008** | 0 (0-1) | 0 (0-1) | 0.686 |
| ICU length of stay (days) | 16 (11-29) | 12 (5-16) | **0.028** | 22 (9-29) | 16 (8-21) | 0.201 |
| Duration of MV (days) | 14(7-25) | 9 (4-15) | 0.053 | 20(9-25) | 12 (5-16) | 0.098 |
| MV free days (days) | 0 (0-0) | 0 (0-15) | **0.035** | 0 (0-0) | 0 (0-13) | **0.019** |
| PaO_2_/FiO_2_ (mmHg) | 122 (86-165) | 130(104-189) | 0.325 | 130 (84-168) | 143 (118-195) | 0.183 |
| Vt (mL/kg PBW) | 6.0 (5.7-6.1) | 5.9 (5.6-6.2) | 0.867 | 6.0 (5.6-6.1) | 6.1 (5.8-6.8) | 0.055 |
| PEEP (cmH_2_O) | 13 (12-15) | 12 (10-15) | 0.217 | 12 (10-15) | 11 (10-14) | 0.183 |
| Driving pressure (cmH_2_O) | 12 (10-15) | 15 (12-16) | 0.125 | 14 (12-17) | 11 (8-14) | **0.038** |
| Crs (mL/cmH_2_O) | 31 (24-40) | 30 (27-33) | 0.572 | 27 (21-33) | 40 (28-49) | **0.012** |
| PaO_2_/FiO_2min_ (mmHg) | 77 (69-101) | 103(85-123) | **0.009** | 94 (73-122) | 108 (82-128) | 0.220 |
| PEEP_max_ (cmH_2_O) | 15 (13-16) | 14 (10-16) | 0.118 | 13 (12-16) | 14 (12-16) | 0.781 |
| Driving pressure _max_ (cmH_2_O) | 17 (15-20) | 16 (13-17) | 0.266 | 17 (13-22) | 14 (11-20) | **0.036** |
| Crs_min_ (mL/cmH_2_O) | 24 (20-27) | 27 (24-28) | 0.071 | 20 (15-29) | 30 (19-40) | **0.005** |
| CI (L/min/m^2^) | 2.93 (2.47-3.63) | 2.40 (1.93-3.43) | 0.081 | 3.28 (2.63-4.72) | 2.73 (2.39-3.56) | 0.114 |
| GEDVi (mL/m^2^) | 753 (651-890) | 621 (562-780) | **0.035** | 752 (532-947) | 685 (593-772) | 0.487 |
| EVLWi (mL/kg) | 19 (14-22) | 14 (13-17) | **0.002** | 19 (15-24) | 13 (10-14) | **<0.001** |
| PVPI | 3.6 (3.0-4.5) | 3.2 (2.6-4.0) | 0.276 | 3.5 (2.4-4.3) | 2.4 (2.1-3.0) | **0.003** |
| GEF (%) | 21 (17-24) | 20 (14-27) | 0.987 | 19 (15-23) | 18 (15-20) | 0.514 |
| CI_min_ (L/min/m^2^) | 2.46 (2.05-2.81) | 2.35 (1.90-2.74) | 0.422 | 2.71 (2.03-3.32) | 2.33 (2.00-2.71) | 0.206 |
| GEDVi_min_ (mL/m^2^) | 648 (556-745) | 604 (507-752) | 0.501 | 623 (532-715) | 588 (544-696) | 0.544 |
| EVLWi_max_ (mL/kg) | 25 (23-28) | 16 (14-18) | **<0.001** | 24 (22-25) | 15 (12-18) | **<0.001** |
| PVPI_max_ | 5.0 (4.4-6.0) | 3.6 (3.1-4.5) | **<0.001** | 4.7 (3.6-5.5) | 3.3 (3.1-4.5) | **<0.001** |
| GEF_min_ (%) | 16 (13-19) | 15 (11-25) | 0.706 | 14 (11-19) | 15 (11-18) | 0.711 |
| NE_max_. (µg/kg/min) | 0.50 (0.17-0.85) | 0.57 (0.08-1.40) | 0.830 | 0.81 (0.50-2.82) | 1.10 (0.53-1.47) | 0.886 |
| Lactate_max_ (mmol/L) | 2.0 (1.6-2.5) | 2.1(1.6-4.6) | 0.520 | 3.0 (2.0-5.9) | 2.7 (2.1-4.2) | 0.711 |
| Fluid balance (mL/day) | 1116(284-1952) | 841 (172-1553) | 0.486 | 809 (171-1398) | 1158 (682-2160) | 0.131 |

Values are expressed as median (interquartile range) or n (%). Bold font indicates statistical significance.

ARDS: acute respiratory distress syndrome; BMI: body mass index; CI: cardiac index; COPD: chronic obstructive pulmonary disease; Crs: respiratory system compliance; ECMO: extracorporeal membrane oxygenation; EVLWi: extravascular lung water indexed for ideal body weight; GEDVi: global end-diastolic volume indexed for body surface; GEF: global ejection fraction; ICU: intensive care unit; MV: mechanical ventilation; NE: Norepinephrine; NMBA: neuromuscular blocking agent; PaO_2_/FiO_2_:ratio of the arterial partial pressure of oxygen over inspired fraction in oxygen; PBW: predicted body weight; PEEP: positive end-expiratory pressure; PVPI: pulmonary vascular permeability index; SAPS: simplified acute physiologic score; SOFA: Sequential Organ Failure Assessment; TPTD: transpulmonary thermodilution; Vt: tidal volume.

#
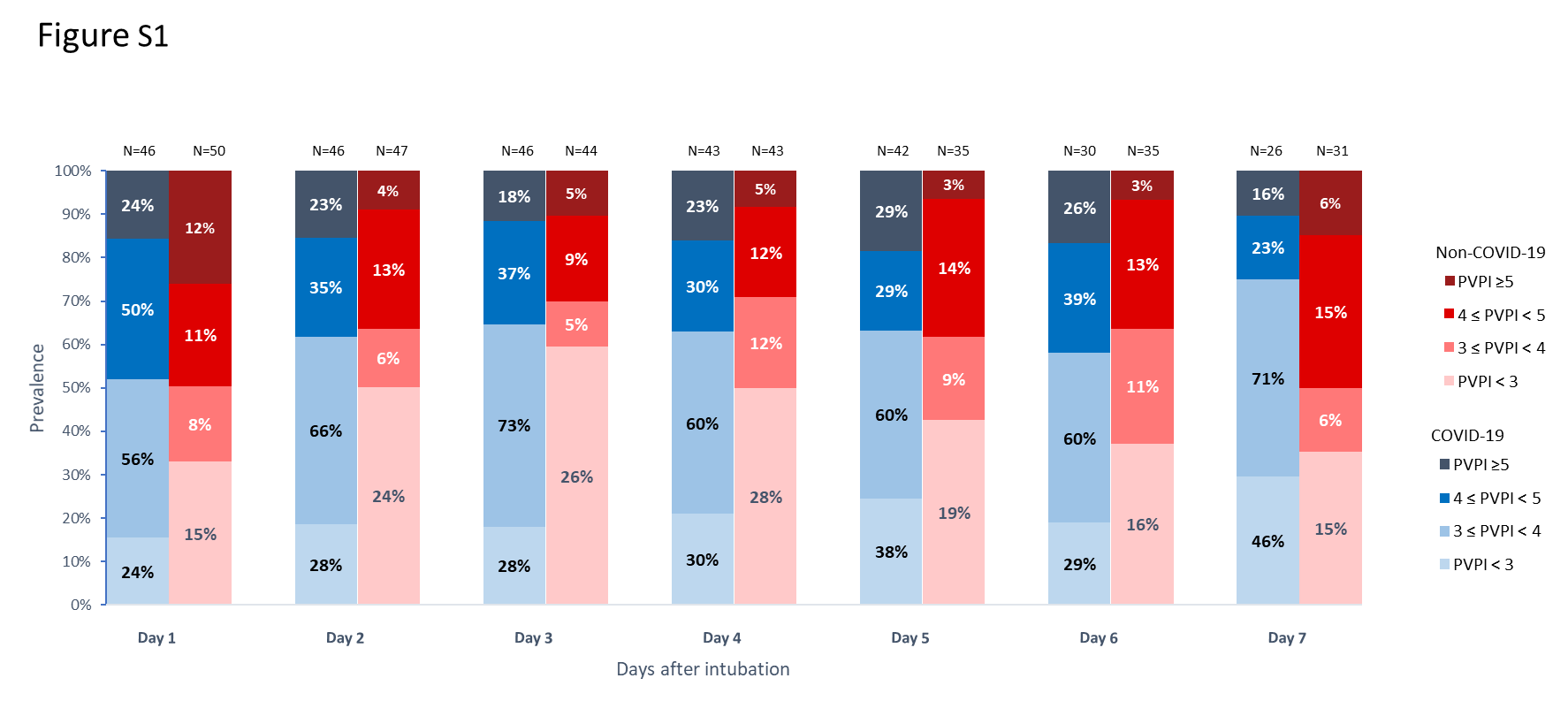
**Figure S1.** Distribution of levels of pulmonary vascular permeability index along with time in COVID-19 and non-COVID-19 acute respiratory distress syndrome.

**
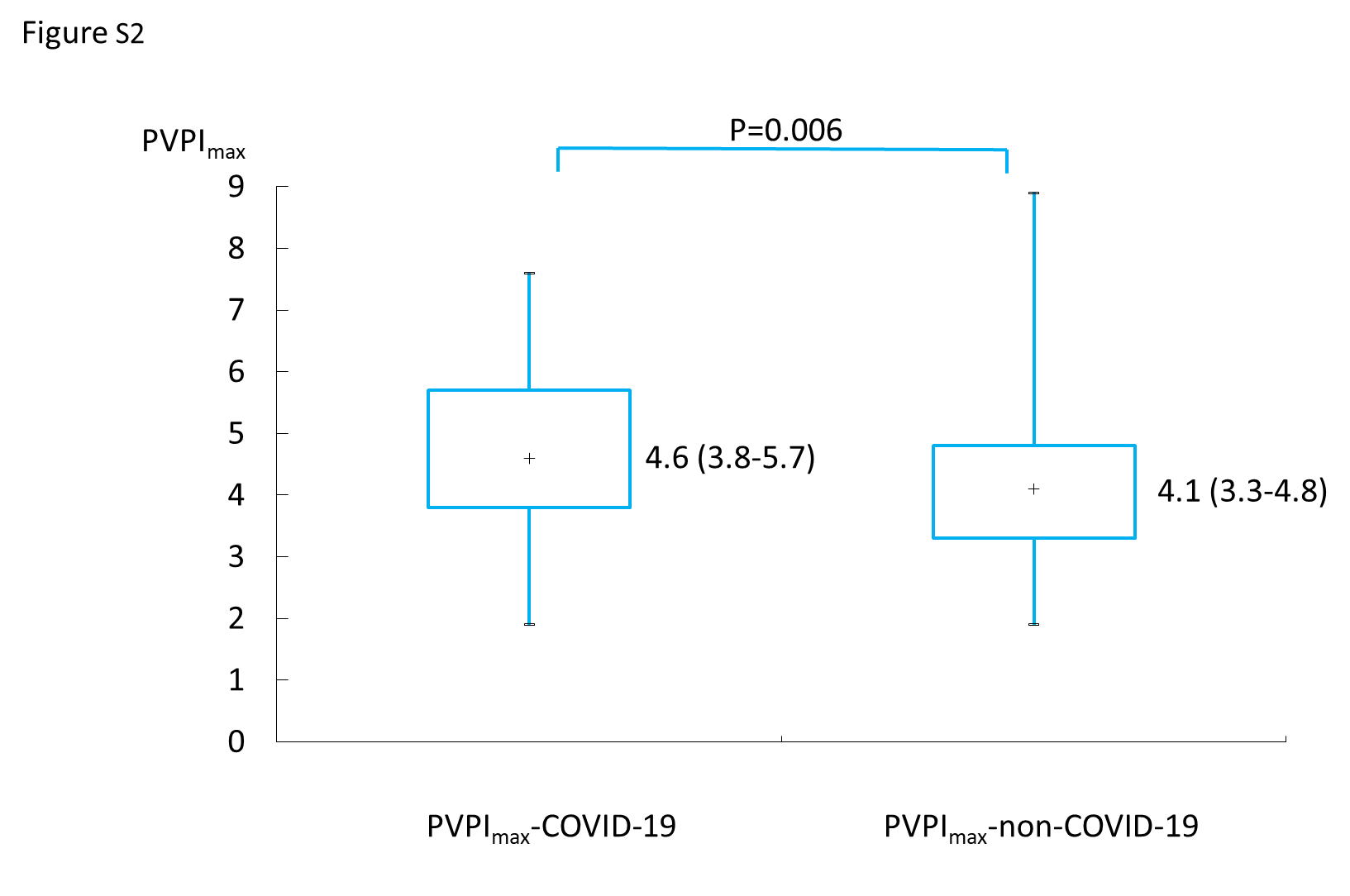
**

# **Figure S2.** Levels of maximum value of pulmonary vascular permeability index in COVID-19 and non-COVID-19 acute respiratory distress syndrome.
